# Prospective Study of Police Officer Spouse/Partners: A New Pathway to Secondary Trauma and Relationship Violence?

**DOI:** 10.1371/journal.pone.0100663

**Published:** 2014-07-02

**Authors:** Susan M. Meffert, Clare Henn-Haase, Thomas J. Metzler, Meng Qian, Suzanne Best, Ayelet Hirschfeld, Shannon McCaslin, Sabra Inslicht, Thomas C. Neylan, Charles R. Marmar

**Affiliations:** 1 Department of Psychiatry, University of California San Francisco, San Francisco, California, United States of America; 2 Mental Health Service, San Francisco Veterans Administration Medical Center, San Francisco, California, United States of America; 3 Department of Psychiatry, New York University, New York, New York, United States of America; 4 Graduate School of Education and Counseling, Lewis and Clark College, Portland, Oregon, United States of America; 5 Dissemination and Training Division, National Center for PTSD, Palo Alto, California, United States of America; Massachusetts General Hospital, United States of America

## Abstract

**Introduction:**

It has been reported that posttraumatic stress disorder (PTSD) is associated with secondary spouse/partner (S/P) emotional distress and relationship violence.

**Objective:**

To investigate the relationships between PTSD, S/P emotional distress and relationship violence among police recruits using a prospective design.

**Methods:**

Two hypotheses were tested in 71 S/Ps: (1) Police officer reports of greater PTSD symptoms after 12 months of police service will be associated with greater secondary trauma symptoms among S/Ps; (2) Greater secondary trauma symptoms among S/Ps at 12 months will be associated with S/P reports of greater relationship violence.

**Methods:**

71 police recruits and their S/Ps were assessed at baseline and 12 months after the start of police officer duty. Using linear and logistic regression, we analyzed explanatory variables for 12 month S/P secondary traumatic stress symptoms and couple violence, including baseline S/P variables and couple violence, as well as exposure and PTSD reports from both S/P and officer.

**Results:**

S/P perception of officer PTSD symptoms predicted S/P secondary traumatic stress. OS/P secondary trauma was significantly associated with both total couple violence (.34, p = .004) and S/P to officer violence (.35, p = .003).

**Conclusions:**

Although results from this relatively small study of young police officers and their S/Ps must be confirmed by larger studies in general populations, findings suggest that S/P perception of PTSD symptoms may play a key role in the spread of traumatic stress symptoms across intimate partner relationships and intimate partner violence in the context of PTSD.

## Introduction

### Secondary Traumatic Stress

The idea that traumatizing events can lead to secondary distress among the close contacts of exposed individuals emerged as a research concept through the work of Charles Figley [1]. The term “secondary trauma” refers to the manifestation of Posttraumatic Stress (PTSD) symptoms among contacts of traumatized individuals, such as family members. Secondary traumatic stress symptoms are typically not as disabling as PTSD, but do include the same types of distress, including avoidance, arousal and intrusive symptom clusters [2]–[5]. Figley describes secondary trauma as arising from an empathic relationship with the traumatized individual through which the close contact is affected by the trauma and manifests symptoms very similar to PTSD.

The current paper focuses on secondary trauma, a concept which should not be confused with related concepts such as vicarious trauma, burnout and compassion fatigue. Vicarious trauma is sometimes used interchangeably with secondary trauma, but is applied most commonly to PTSD symptoms among healthcare providers working with traumatized individuals [6]. Burnout refers to a syndrome of exhaustion, cynicism/detachment and sense of inefficacy related to interpersonal job stressors, which can include work with traumatized individuals [7]. Compassion fatigue has been described as a specific type of secondary trauma stress related to the care-giver “burnout” [8].

### PTSD and Spouse/Partners (S/Ps)

In cross sectional analyses, trauma exposure, alone, as well as PTSD, and S/P perception of PTSD symptoms are associated with secondary traumatic stress among spouses [2], [3], [9]–[11]. Individuals who have PTSD also have more violent marriages than those without PTSD. Not only are individuals with PTSD at higher risk of violence toward their partners, but they are also more likely to receive violence from their partners [5]. These findings have been confirmed in studies of military personnel and their S/Ps, including: Renshaw, 2011; Goff, 2007; Riggs, 1998; Taft, 2007; McDonald, 1999; Teten, 2010 [12]–[16]. Relative to military populations, studies of both male and female civilians have found a variable impact of PTSD on intimate relationship satisfaction and intimate partner violence (IPV), including both non-significant and [17] and significant effects [18]–[21]. The one tangentially related study of police officers of which we are aware, examined gender differences in compassion fatigue, PTSD symptoms and relationship satisfaction between male and female police detectives who investigate sexual offenses against children. The study found that PTSD symptoms were correlated with compassion fatigue, and that open relationship communication was associated with lower compassion fatigue for men but higher fatigue for women [22].

The cross sectional nature of previous studies does not allow determination of whether S/P distress and relationship difficulties predated exposure/PTSD or were a product of them. Without pre-exposure prospective data on both spouses, the nature of the links between trauma exposure, intimate partner secondary traumatic stress and relationship difficulties can only be hypothesized.

To address this knowledge gap, we examined data from a prospective study of uniformed services personnel known to have high exposure to traumatic stressors: police recruits in large urban settings. The study’s longitudinal design with repeated measurements of both officers and partners provides an opportunity to determine the directionality of the relationship between S/P secondary trauma and intimate partner violence in the context of traumatic exposure. Two hypotheses were tested in 71 S/Ps: (1) Greater PTSD symptoms among officers after 12 months of police service will be associated with greater secondary trauma symptoms among S/Ps; (2) Greater secondary trauma symptoms among S/Ps at 12 months will be associated with S/P reports of greater difficulties on measures of relationship violence.

## Methods

### Participants

This study examines the impact of secondary trauma symptoms on intimate partners and intimate partner relationships among 71 partner participants collected as part of a larger, multi-site longitudinal study of U.S. urban police officer stress and health, approved by the University of California Human Subjects Committee and Institutional Review Board. The parent study was designed to assess the predictors and neurobiological correlates of PTSD following trauma exposure using a longitudinal prospective analysis of new recruits and their partners with multidimensional baseline and outcome measures. All participants provided written informed consent. Consent procedures were also approved by the University of California Human Subjects Committee and Institutional Review Board. Participants were recruited from four urban police departments, the New York Police Department (NYPD) and three departments in the San Francisco Bay Area (Oakland, OPD; San Francisco, SFPD; and San Jose, SJPD) during police academy training. Academy trainees were introduced to the study through an in-person presentation made by study personnel during academy training classes. This presentation included the distribution of two letters, one from the commissioner or police chief of the affiliated department and one from the study team. Included with the approach letters was a description of the study procedures, a contact number, and a participation form with the option to be contacted by the study team. Only those trainees who were combat veterans at the time of academy training or who had prior experience in law enforcement or emergency services occupations were excluded from participating in the study. Study participants were provided with forms to give to their S/Ps in order to enroll in the S/P study. Data is available upon request.

### Procedures

#### Baseline Assessment

The baseline assessment of both officers and their S/Ps was conducted while the officer was in training at the police academy. The baseline assessment included a self-report questionnaire package for both officers and S/Ps. Baseline measures were: (1) officer demographic data, (2) S/P depression (Beck Depression Index, BDI), (3) S/P report of relationship conflict (Conflict Tactics Scale, CTS) and (4) S/P hostility (Symptom Checklist-90-Revised (SCL-90-R-hostility). See below for descriptions of measures.

#### Follow-up Assessment

Participating officers were re-contacted at 12 months after commencing active police duty. They were administered a self-report questionnaire packet assessing domains of psychological functioning. At the same time point, S/Ps completed self-report questionnaires assessing psychological functioning including secondary traumatization and relationship adjustment. For the purposes of this study, “secondary trauma” symptoms (Modified Secondary Trauma Questionnaire (MSTQ)) were defined as PTSD symptoms reported by S/Ps in response to officers’ critical incident exposure (criterion A). Twelve month officer measures included: (1) officer report of their exposure to critical incidents (Critical Incident History Questionnaire (CIHQ)) and (2) officer report of their PTSD symptoms (Mississippi Combat Scale-Civilian Version (MCS-CV)). Twelve month S/P measures included: (1) secondary trauma (MSTQ), (2) conflict (CTS), (3) depression (BDI), (4) S/P ratings of officer exposure to critical incidents (CIHQ), and (5) S/P ratings of officers’ PTSD symptoms (MCS-CV). See below for descriptions of measures.

### Measures (Appendix S1)

#### Outcome Variables

*Modified Secondary Traumatic Stress Questionnaire (MSTQ)* [23]. The 20-item Secondary Trauma Scale (STS) was originally developed in a sample of students and mental health professionals [24]. In a subsequent follow-up study, the STS was shortened to 18 items by dropping the two items on experiencing horror and disturbing recollections [25]. Each S/P was asked to list the three most distressing events that the officer had experienced in the line of duty in the past six months. S/Ps were asked to describe their own symptoms relating to officers’ “duty-related distressing experiences.” MSTQ questions correspond to the major PTSD criteria listed in the DSM-IV. Ratings are made on a 5-point Likert scale ranging from “Rarely/never” (1) to “Very often” (5). The 18 items are summed to generate a total score that ranges from 18–90, with scores of 45 or higher indicative of problematic symptoms of intrusion and avoidance that should be of substantive clinical concern [26]. The STS/MSTQ has been shown to have good internal consistency, as represented by Cronbach’s alpha values ranging from 0.75 [24] to 0.89 [26]. Its construct validity is supported by its strong (r = 0.61) correlation with the Beck Anxiety Inventory [26] and moderate correlations (r = 0.33–0.49) with measures of primary trauma [24]. The MSTQ was administered to S/Ps at 12 months. The Cronbach’s alpha in this sample was 0.92.

*Conflict Tactics Scale (CTS)* [27]. The CIHQ is a 34-item self- report measure assessing cumulative exposure to critical incidents. Participants tabulate the number of times (frequency of exposure) that they have personally experienced each of the 34 critical incidents in the line of duty. The total cumulative exposure score is derived by summing the frequency of incident exposure across all items. The CIHQ was log transformed in order to reduce extreme skew and account for the effect of repeated exposures. For example, the difference between the effect of experiencing an event once versus twice is expected to be greater than the difference between the effect of 50 or 51 events. Moreover, more distressing events tend to be experienced with low frequency, while high frequency events tend to be less distressing [28]. We obtained a CIHQ self-report from officers at 12 months. We also obtained a modified CIHQ in which S/Ps were asked to report on the critical incidents experienced by the officer with whom they were in a relationship.

#### Explanatory Variables

*Beck Depression Inventory (BDI)* [29]. The Second Edition (BDI-II) is a 21-item instrument intended to assess the existence and severity of depression symptoms over the past two weeks. Each of the 21 items corresponding to a symptom of depression is summed to give a single score for the BDI-II. There is a four-point scale for each item ranging from 0 to 3. The BDI has been used for 35 years to identify and assess depressive symptoms, and has been reported to be highly reliable regardless of the population. It has a high coefficient alpha (0.91) its construct validity has been established, and it is able to differentiate depressed from non-depressed patients [16]. The BDI was administered to S/Ps at baseline and at 12 months. The Cronbach alpha in this sample was 0.861.

*Critical Incident History Questionnaire (CIHQ)* [28]. The CIHQ is a 34-item self- report measure assessing cumulative exposure to critical incidents. Participants tabulate the number of times (frequency of exposure) that they have personally experienced each of the 34 critical incidents in the line of duty. The total cumulative exposure score is derived by summing the frequency of incident exposure across all items. The CIHQ was log transformed in order to reduce extreme skew and account for the effect of repeated exposures. For example, the difference between the effect of experiencing an event once versus twice is expected to be greater than the difference between the effect of 50 or 51 events. Moreover, more distressing events tend to be experienced with low frequency, while high frequency events tend to be less distressing. [28] We obtained a CIHQ self-report from officers at 12 months. We also obtained a modified CIHQ in which S/Ps were asked to report on the critical incidents experienced by the officer with whom they were in a relationship.

*Mississippi Combat Scale-Civilian Version (MCS-CV)* [23]. The MCS-CV is a 35-item measure that assesses PTSD-related symptoms of intrusion, avoidance, hyperarousal, and other difficulties since the time *of* critical incident or trauma exposure. The civilian version has been used to assess PTSD symptoms in non-veteran controls as part of the National Vietnam Veterans Readjustment Study [30] and in emergency services personnel following a disaster [31], [32]. Responses are recorded in a 5 point Likert scale. Items 2, 6, 11, 17, 19, 22, 24, 27, 30, and 34 are reverse coded. Items are summed to obtain the total MCS-CV score. For this study, participants were asked to report symptoms since beginning police service (12 months prior). S/Ps also completed the MCS-CV, reporting symptoms of the officers with whom they were in a relationship.

*Symptom Checklist-90-R (SCL-90-R)* [33]. The Symptom Checklist-90-R (SCL-90-R) is a self-report instrument is used to evaluate a broad range of psychological problems and symptoms of psychopathology. Reporters indicate on a Likert scale ranging from 0 (not at all) to 4 (extremely) the extent to which they have experienced various psychological symptoms in the past 7 days. There are nine clinical subscales [34]. The SCL-90-R was administered to S/Ps at baseline and 12 months. The GSI scale had a Cronbach’s alpha of 0.96 in this sample. For the purposes of this study, we used the SCL-Hostility subscale, given the literature supporting an association between relationship violence and hostility, in the context of traumatic stress [14], [18], [35]. We used the BDI (above) as a measure of depression symptoms rather than the SCL-90 depression subscale for consistency with outcome scores in prior publications [36].

## Data Analysis

Fifty-seven of the 71 participants (80%) had complete data on all model predictors. While only 6.2% of the data were missing, 14 participants (20%) would need to be dropped from complete-case regression analyses. Missing data indicators tended to correlate with values of non-missing variables, indicating that data are not missing completely at random (MCAR; [37]). We therefore conducted a multiple imputation of missing data under the assumption of missingness at random (MAR), meaning that missing data values are considered random conditional on values of observed non-missing data. We employed the method of chained equations [38], as implemented in the “mi” suite in Stata 13 [39]. This is an iterative procedure in which a regression model is constructed for missing values on each variable, and missing data are modeled with an appropriate regression equation (linear regression for continuous variables, logistic regression for binary variables, and ordinal logistic regression for ordered categorical variables). This method does not require that all variables share a common multivariate distribution, which is unlikely to hold with disparate types of variables. Candidate variables for each predictor model comprised the full set of 12 predictors and outcomes, plus any repeated scores on variables (e.g., BDI at 1 year was included in the model for BDI at baseline). Candidate predictors showing a Pearson correlation of at least r = .20 with the imputation variable in the complete data set, were included in the initial imputation model. Variables were removed from each model via backward selection, with a significance level of p = .10. Final imputation models contained from one to three predictors. Continuous variables were zero-skew log transformed (“lnskew0” command in Stata 13) before imputation, and then back-transformed to original units for analysis after imputation. Binary variables were imputed with logistic regression, and the 5-level categorical Education Level variable was imputed using ordinal logistic regression. Twenty imputation data sets were produced by randomly selecting data from the posterior distribution of each imputation model. Analyses were conducted using the “mi estimate” estimation commands in Stata, which adjust coefficients and standard errors for the variability among imputations according to Rubin’s combination rules [40].

Pearson correlations among the variables of interest were evaluated. In order to estimate the association between secondary trauma symptoms among S/P and PTSD symptoms among officers, we fit a multiple linear regression model to the data, specifying secondary S/P trauma (MSTQ) at 12 months as the dependent variable. Covariates included baseline S/P depression (BDI-II) and hostility (SCL-Hostility), 12 month officer and S/P report of critical incidents and 12 month officer and S/P report of PTSD.

Two logistic regressions were run with S/P report of total couple violence (CTS subscale) at 12 months and S/P report of partner to officer violence as the dependent variables (CTS subscale). Twelve month violence scores were converted to a categorical variable (assigning the value of “1” to 12 month violence subscale scores greater than 0 and assigning a value of “0” to 12 month violence subscale scores of 0). Models included baseline S/P ratings of couple violence, officers’ trauma exposure (CIHQ) (officer and S/P ratings), report of officers’ PTSD symptoms (officer and S/P ratings) and S/P secondary trauma symptoms (MSTQ).

## Results

### Participants

The majority of police recruits were male and the majority of S/Ps were female (Table 1). Approximately 50% of police recruits and S/Ps were white. 10–20% were black, Hispanic or Asian in both recruits and S/Ps. For both recruits and S/Ps, the majority held a bachelor’s degree/completed 4 years of undergraduate study. The majority of recruits earned 30,000–50,000 usd/year and majority of S/P earned 15,000–30,000 usd/year. At baseline, S/Ps reported low levels of depression and hostility and approximately one-quarter reported couple violence (Table 1). At 12 months, the average S/P secondary traumatic stress score was 26.8, corresponding to mild symptoms (Table 1) and five S/Ps (7%) met the threshold for secondary trauma (45) on the MSTQ [24], [26]. As expected, the average officer MCS-CV symptom score at 12 months (59.82, Table 1) was below that of PTSD patients (130) and psychiatric populations (86) [41]. No officers met criteria for full PTSD disorder at 12 months.

**Table 1:** Sample Characteristics.

	Police Recruit *M* or *n* (SD or %)	S/P *M* or *n* (SD or %)
Female	5 (8%)	62 (94%)
Male	61 (92%)	4 (6%)
Age	29.5 (5.2)	27.9 (5.8)
Race		
*White*	34 (53%)	33 (50%)
*Black*	7 (11%)	6 (9%)
*Asian*	10 (16%)	11 (17%)
*Hispanic*	10 (16%)	13 (20%)
*Black-Native American*		1 (2%)
*Asian-White-Hispanic*		1 (2%)
*White-Hispanic*		1 (2%)
*Other*	3 (5%)	
Male-male couples	0	
Female-female couples	1	
Education		
*<High School*	0 (0%)	2 (4%)
*High School/GED*	7 (10%)	15 (27%)
*2 years college/AA degree*	23 (33%)	11 (20%)
*4 years college/bachelor’s degree*	32 (46%)	22 (40%)
*Master’s Degree*	7 (10%)	5 (9%)
*Doctoral Degree*	1 (1%)	0 (0%)
Income		
*Up to 15* *K*	1 (1%)	8 (15%)
*15,001–30* *K*	1 (1%)	14 (25%)
*30,001–50* *K*	27 (39%)	2 (4%)
*50,001–70* *K*	21 (30%)	13 (24%)
*70,001–90* *K*	12 (17%)	13 (24%)
*More than 90* *K*	8 (11%)	5 (9%)
Outcome Variables		
*Secondary Traumatic Stress (MSTQ)*		26.8 (11)
*Total Couple Violence (CTS)*		
*Yes*		19 (26.8%)
*No*		52 (73.2%)
*S/P to Recruit Violence (CTS)*		
*Yes*		17 (23.9%)
*No*		54 (76.1%)
Explanatory Variables		
*Depression (BDI)*		6.43 (6.17)
*Officer PTSD Symptoms, S/P Report*		42.2 (7.35)
*Officer PTSD Symptoms, Officer Report*		59.82 (9.58)
*Hostility (SCL-Hos)*		0.35 (.33)

### Correlations among study variables (Table 2)

At 12 months, S/P reported a mean secondary trauma (MSTQ) score of 26.8 (SD = 11) which as significantly correlated with S/P baseline depression (Pearson’s r = .55) and hostility (r = .23), as well as 12 month S/P report of officer’s PTSD (Pearson’s rho = .64) and 12 month violence measures including total couple violence (.34) and S/P toward officer violence (r = .35). There was no significant correlation between officer report of PTSD symptoms and S/P secondary trauma. The only demographic variable to show a relationship with secondary trauma was education level, with higher education level associated with higher secondary trauma scores (r = .34, p<.01). There was not a significant correlation between officer and S/P report of officer PTSD symptoms at 12 months. Total couple violence and S/Ps’ violence toward officers were significantly correlated with S/P baseline hostility (r = .28, .31, respectively), baseline violence (r = .78, .75), S/P report of officer PTSD at 12 months (Pearson’s rho = .33, .33) and S/P secondary trauma at 12 months (r = .33, .38).

**Table 2:** Pearson correlations among variables of interests.

Measure	1	2	3	4	5	6
**1. S/P secondary trauma at 12 month**						
**2. Police PTSD symptom at 12 month**	−0.04					
**3. S/P baseline depression**	0.55***	−0.15				
**4. S/P perception of police PTSD** **symptom at 12 month**	0.64***	0.09	0.24			
**5. S/P baseline hostility**	0.30*	−0.10	0.49***	0.09		
**6. S/P towards police violence at 12 month**	0.35**	0.06	0.15	0.40***	0.35**	
**7. Total violence at 12 month**	0.34**	0.06	0.09	0.48***	0.29**	0.93***

*Note*. *p≤.05,

**p≤.01,

***p≤.001.

### Predictors of Secondary Trauma among Spouse/Partners of Police Officers (Table 3)

The dependent variable is the total S/P secondary traumatic stress symptom MSTQ score at 12 months. S/P education, baseline depression symptoms and reports of officer’s PTSD symptoms at 12 months were significantly and directly associated with secondary traumatic stress among S/Ps at 12 months. The final model accounted for 44.7% of the variance in secondary trauma symptoms among S/Ps (adjusted R squared).

**Table 3:** Linear Regression Model Predicting S/P Secondary Trauma at 12 months (*N* = 71).

Predictors	*β* [95% C.I.]
S/P Education Level	.25* [.05, .45]
S/P Hostility at Baseline	.03 [−.17, .23]
S/P Depression at Baseline	.35** [.11, .59]
Officer Exposure Officer Report	−.15 [−.37, .08]
Officer Exposure S/P Report	.04 [−.01, .08]
Officer PTSD symptoms officer report	−.04 [−.24, .16]
Officer PTSD symptoms S/P Report	.45*** [.22, .67]
**Adjusted R^2^**	.45

*Note*. *p≤.05,

**p≤.01,

***p≤.001.

### Predictors of Violence (Table 4)

Nested logistic regression was used to evaluate the predictors of total couple violence and S/P to officer violence (Table 4). In both models after entering baseline violence, none of the covariates were significant at any step until S/P perception of officer PTSD symptoms was added in the final step. In the two final models both baseline violence and S/P perception of officer PTSD were significant predictors of S/P to officer violence.

**Table 4:** Logistic regression model predicting couple violence at 12 months (*N* = 71).

Predictors: All Couple Violence	Odds Ratio [95% C.I.]	Predictors: S/P to Officer Violence	Odds Ratio [95% C.I.]
1. Baseline Couple Violence	16.9** [2.5, 114.9]	1. Baseline S/P to Officer Violence	18.6** [2.6, 133.1]
2. Officer Trauma Exposure Report	1.19 [0.50, 2.80]	2. Officer Trauma Exposure Report	0.91 [0.39, 2.10]
3. S/P report of officer exposure	1.10 [0.45, 2.65]	3. S/P report of officer exposure	1.50 [0.64, 3.50]
4. Officer Report of Officer PTSD	1.03 [0.44, 2.40]	4. Officer Report of Officer PTSD	0.79 [0.32, 1.98]
5. S/P Report of Officer PTSD	2.86* [1.13, 7.25]	5. S/P Report of Officer PTSD	2.80* [1.05, 2.99]
6. 12 month Secondary Trauma	1.53 [0.59, 3.99]	6. 12 month Secondary Trauma	1.30 [0.53, 3.18]

*Note*. Baseline violence (couple violence and S/P to officer) is coded yes/no, all other predictors are standardized continuous variables; *p≤.05, **p≤.01, ***p≤.001.

## Discussion

In our prospective analysis of S/Ps of police officers exposed to duty-related traumatic stress, we found average S/P secondary traumatic stress scores to be relatively low at 12 months (26.8), corresponding to mild symptoms [42]. S/P perception of officer PTSD symptoms at 12 months was significantly associated with S/P secondary traumatic stress and couple violence. Variables which have been associated with secondary traumatic stress and couple violence in other studies, such as trauma exposure and officer PTSD symptoms, were not significant when S/P perception of officer PTSD was added to the models.

Hypothesis one, postulating that officer report of greater PTSD symptoms would be associated with greater secondary trauma symptoms among S/Ps, was not confirmed. Officer and S/P report of officer PTSD symptoms were moderately correlated, consistent with prior findings. [43] When modeled to predict secondary traumatic stress among S/Ps, only baseline S/P depression and S/P perception of PTSD symptoms in the officer were significant. The latter finding is consistent with the recent work of Renshaw and colleagues, identifying spousal perception of PTSD symptoms as a mechanism for development of psychological distress among spouses of veterans which mediates the effect of veteran report of PTSD symptoms. Renshaw uses an attributional model to explain how spousal distress may correlate with PTSD symptoms. Noting that spousal distress increases with greater discrepancy between spousal and veteran report of PTSD symptoms and with S/P perception of avoidance/numbing symptoms, Renshaw and colleagues postulate that increased difficulty attributing behaviors to PTSD leads to higher spousal distress [44]. We observed a discrepancy in report of PTSD symptoms with officers reporting higher PTSD symptoms than perceived by their S/Ps (Table 1). The S/P-officer PTSD symptom discrepancy found in this study may partly explain why S/P distress was not associated with officer PTSD (Hypothesis 1). According to the attributional model used by Renshaw, it is, in fact, the lack of knowledge of officer PTSD that is correlated with high S/P distress.

Hypothesis two, postulating that greater secondary trauma would be associated with greater S/P reports of relationship violence, was confirmed. In correlation analysis (Table 2), S/P secondary traumatic stress was significantly associated with total couple violence and S/P to officer violence. In regression analyses of violence (Table 4), secondary traumatic stress was not a significant predictor in the final model. The latter finding is not surprising given that S/P perception of officer PTSD was included in the model and is highly associated with secondary traumatic stress. Significant predictors of both total couple violence and S/P to officer violence included baseline violence and S/P perception of officer PTSD symptoms. In contrast to previous findings, exposure variables and officer report of their own PTSD symptoms failed to predict total couple violence or S/P violence at 12 months.

In summary, the findings of this study are consistent with current attributional theories of S/P distress in the setting of PTSD and build on them using a prospective design. This study provides support for the idea that S/P perception of PTSD symptoms is highly linked to S/P distress, controlling for baseline S/P factors, and adds to our growing understanding of the mechanism by which PTSD leads to intimate partner violence.

## Limitations

This study was part of a larger longitudinal cohort study designed to detect risk factors for PTSD following trauma exposure. As such, it was not powered specifically to address the questions in this sub-analysis. It is possible that the study was underpowered for detection of a significant association between officer PTSD and S/P secondary traumatic stress, explaining a negative finding (Hypothesis 1). Second, in this study, the measures of violence were taken from S/Ps and not from the officers. It is possible that, if relationship measures were taken from officers, they would have a stronger association with officer reports of PTSD symptoms. Third, this is a sample of young, healthy, police academy officers, who are relatively resilient and are not representative of the general population, necessitating caution in generalizing our findings. On average, officers and their S/Ps had low levels PTSD and secondary trauma symptomatology. Although symptoms are generally on a continuum with diagnoses in the field of trauma studies, it is possible that the results we present here are not applicable to populations with higher level of symptoms/PTSD diagnosis. Fourth, the officer sample is predominantly male and the S/Ps are predominantly female. The gender distribution did not permit us to model the impact of trauma/secondary trauma symptoms on a partnership in which the trauma exposed individual was female, or S/P was male. Finally, the study examines S/P secondary traumatic stress related to police duty and does not assess S/P baseline trauma symptoms attributed to other stressors. It is possible that if baseline trauma symptoms had been measured and controlled in the analysis, a stronger relationship would have emerged between police PTSD symptoms and S/P secondary traumatic stress.

## Conclusions

This study contributes to the field of PTSD research by broadening our understanding of how traumatic stress symptoms impact intimate partner relationships. Unlike previous studies, we measured S/P perception of PTSD symptoms and controlled for baseline variables prior to trauma exposure in our models for S/P secondary trauma and couple violence. If these findings can be replicated in general populations, they suggest that perception of a partner’s PTSD symptoms is linked to destructive emotional and interpersonal conditions, including secondary trauma symptoms and couple violence.

## Supporting Information

Appendix S1 Measures used in the study.
(DOCX)
