# Supplementary material for: Prospective Study of Police Officer Spouse/Partners: A New Pathway to Secondary Trauma and Relationship Violence?
Source: PLoS One. 2014 Jul 2;9(7):e100663. doi: 10.1371/journal.pone.0100663 (PMC4079247; doi:10.1371/journal.pone.0100663)
Supplement: Appendix S1 — Measures used in the study. (DOCX) [file pone.0100663.s001.docx]

**Appendix A: Questionnaires**

**Beck Depression Index (BDI):** Harcourt Assessment, Inc. administers the rights for the Beck scales under contract from Dr. Beck. This tool can be purchased from them. The cost is $75 which includes the manual and 25 record forms. Additional forms are $40 for 25 or $145 for 100. Harcourt Assessment, Inc. 19500 Bulverde Road San Antonio, Texas 78259 Phone: 1-800-211-8378 Fax: 1-800-232-1223 Email:  abeck@mail.med.upenn.edu; Website:  www.harcourtassessment.com

**Conflict Tactics Scale (CTS**): Questionnaire and scoring method available from Western Psychological Services (WPS): http://www.wpspublish.com

**Critical Incident History Questionnaire (CIHQ):**

INSTRUCTIONS: Below is a list of critical incidents that police officers may experience at

sometime during their career. Please read each item and in the left-hand column, give your

best estimate of the number of times that your partner has personally experienced that

incident in the line of duty SINCE BECOMING A POLICE OFFICER. Next, in the right-hand

column, please give your opinion about how difficult it would be for police officers in

general to cope with each type of incident, NOT how difficult you think it would be for your

partner personally. Please provide your opinion of the difficulty of each incident, even if you

do not believe your partner has ever experienced it.

**Response options (frequency):**

Write in the # 10-20 21-50 51+

if from 0-9

Response options (difficulty):

Not at all a little bit moderately quite a bit extremely

1. Being seriously injured intentionally.

3. Being present when a fellow officer was killed intentionally.

4. Being present when a fellow officer was seriously injured intentionally.

5. Being present when a fellow officer was seriously injured accidentally.

2. Being seriously injured accidentally.

6. Being present when a fellow officer was killed accidentally.

7. Being seriously beaten in the line of duty.

8. Being taken hostage in the line of duty.

9. Receiving serious threats towards loved ones as retaliation for their police work.

10. Being shot at in the line of duty.

11. Being threatened with a gun in the line of duty.

12. Being threatened with a knife or another weapon in the line of duty.

13. Being trapped in a potentially life-threatening situation in the line of duty.

14. Being exposed to serious risk of AIDS or other life-threatening diseases in the line of duty.

15. Having his or her life threatened by an aggressive an [sic] dangerous animal in the line of duty.

17. Having to kill or seriously injure someone in the line of duty.

16. Being exposed to a life-threatening toxic substance in the line of duty.

18. Having to shoot at someone in the line of duty, without injuring them.

19. Making a mistake that led to the serious injury or death of a fellow officer.

20. Making a mistake that led to the serious injury or death of a bystander.

21. Being involved in a high speed chase where lives were in danger.

22. Seeing someone dying.

23. Encountering the body of someone recently dead.

24. Encountering a decaying corpse.

25. Encountering a mutilated body or human remains in the line of duty.

26. Making a death notification.

27. In the line of duty, encountering a child who had been sexually assaulted.

28. In the line of duty, encountering an adult who had been sexually assaulted.

29. In the line of duty encountering a child who had been badly beaten.

30. In the line of duty encountering an adult who had been badly beaten.

31. In the line of duty encountering a child who was severely neglected or in dire need of medical attention because of neglect.

32. In the line of duty seeing animals that had been severely neglected, intentionally injured, or killed.

33. Having his or her life endangered in a large-scale man-made disaster in the line of duty.

34. Having his or her life endangered in a large-scale natural disaster in the line of duty.

If your partner has experienced a critical incident in the line of duty that does not fit into

any of the categories mentioned above, please describe the event(s) in the space below.

35. Event:

36. Event:

Scoring Method: The total cumulative exposure score was derived by summing the frequency of incident exposure across all items.

**Mississippi Scale for (MSC):** This measure was created by staff at the VA National Center for PTSD. To obtain this scale complete the online request form: http://www.ncptsd.org; or email: ncptsd@ncptsd.org. Information on measures is available to everyone. However, the assessment tools themselves can only be distributed to qualified mental health professionals and researchers. Scoring Method: Each item is rated on a 5-point Likert scale, with items 2, 6, 11, 17, 19, 22, 24, 27, 30, and 34 scored in the reverse order (i.e. subtract their rating from 6). All the items are than added to obtain a total score on the scale.

**Modified Secondary Trauma Questionnaire (MSTQ):**

Consider the most distressing experiences that have happened to your spouse or partner in the line of duty over the past six months.

Briefly described what you consider to be the 3 most distressing experiences that your spouse or partner has experienced in the line of duty of the past 6 months:______________________________________________________________

If you can't think of any distressing experience, please put a check here:

For the items below, fill in the oval that best describes how you think and feel.

Response options:

Rarely/Never At Times Not Sure Often Very Often

1. I force myself to avoid certain thoughts or feelings that remind me of my partner's duty-related distressing experiences.

2. I find myself avoiding certain activities or situations because they remind me of my partner's duty-related distressing experiences.

3. Over the past 6-months, I have difficulty falling or staying asleep.

4. Over the past 6-months, I startle easily.

5. I have vivid unwanted images or memories related to my partner's duty-related distressing experiences.

6. I am frightened by things that my partner has told me about his/her police work.

7. I experience troubling dreams that I believe are related to my partner's duty-related distressing experiences.

8. I experience intrusive, unwanted thoughts about my partner's duty-related distressing experiences.

9. I am losing sleep due to thoughts of my partner's duty-related distressing experiences.

10. I am concerned that I might be negatively affected by my partner duty-related distressing experiences.

11. I have felt "on edge" and distressed and this may be related to thoughts about my partner's duty-related distressing experiences.

12. Over the past 6-months, I have tried to avoid dealing with my partner.

13. I have difficulty recalling specific aspects and details of my partner's duty-related distressing experiences.

14. Over the past 6-months, I find myself losing interest in activities that used to bring me pleasure.

15. Over the past 6-months, I find it increasingly difficult to have warm and positive feelings for others.

16. Over the past 6-months, I find that I am less clear and optimistic about my future life than I once was.

17. Over the past 6-months, I have had some difficulty concentrating.

18. When I think about what it would be like to have experienced what my partner has while on duty, I feel threatened and vulnerable.

Scoring Method: The 18 items are summed to generate a total score that ranges from 18-90, with scores of 45 or higher indicative of problematic symptoms of intrusion and avoidance that should be of substantive clinical concern.

**Symptom Checklist-90-Revised (SCL-90-R-hostility):** Questionnaire and scoring method available from Pearson: http://www.pearsonclinical.com/education/products
